# Familial autoimmunity in patients with idiopathic inflammatory myopathies

**DOI:** 10.1111/joim.13573

**Published:** 2022-10-10

**Authors:** Weng Ian Che, Helga Westerlind, Ingrid E. Lundberg, Karin Hellgren, Ralf Kuja‐Halkola, Marie E. Holmqvist

**Affiliations:** ^1^ Clinical Epidemiology Division Department of Medicine, Solna Karolinska Institutet Stockholm Sweden; ^2^ Division of Rheumatology Department of Medicine, Solna Karolinska Institutet Stockholm Sweden; ^3^ ME Gastro, Derm and Rheuma Theme Inflammation and Aging Karolinska University Hospital Stockholm Sweden; ^4^ Department of Medical Epidemiology and Biostatistics Karolinska Institutet Stockholm Sweden

**Keywords:** autoimmune diseases, familial autoimmunity, idiopathic inflammatory myopathies, shared familial susceptibility, shared genetic susceptibility

## Abstract

**Background:**

Familial associations can be indicators of shared genetic susceptibility between two diseases. Previous data on familial autoimmunity in patients with idiopathic inflammatory myopathies (IIM) are scarce and inconsistent.

**Objectives:**

To investigate which autoimmune diseases (ADs) may share genetic susceptibility with IIM, we examined the familial associations between IIM and different ADs.

**Methods:**

In this Swedish population‐based family study, we assembled 7615 first‐degree relatives (FDRs) of 1620 patients with IIM and 37,309 relatives of 7797 matched individuals without IIM. Via register linkages, we ascertained rheumatoid arthritis, other rheumatic inflammatory diseases (RIDs), multiple sclerosis, inflammatory bowel diseases (IBD), type 1 diabetes mellitus, autoimmune thyroid diseases (AITD), coeliac disease (CeD) and myasthenia gravis among the FDRs. We estimated the familial association between IIM and each AD using conditional logistic regression and performed subgroup analyses by kinship.

**Results:**

Patients with IIM had significantly higher odds of having ≥1 FDR affected by other RIDs (adjusted odds ratio [aOR] = 1.40, 95% confidence interval [CI] 1.11–1.78) and greater odds of having ≥2 FDRs affected by CeD (aOR = 3.57, 95% CI 1.28–9.92) compared to the individuals without IIM. In the analyses of any FDR pairs, we observed familial associations for other RIDs (aOR = 1.34, 95% CI 1.14–1.56), IBD (aOR = 1.20, 95% CI 1.02–1.41), AITD (aOR = 1.10, 95% CI 1.02–1.19) and CeD (aOR = 1.37, 95% CI 1.08–1.74) while associations for other ADs were not statistically significant.

**Conclusion:**

The observed familial associations may suggest that IIM shares genetic susceptibility with various ADs, information that may be useful for clinical counselling and guiding future genetic studies of IIM.

## Graphical abstract

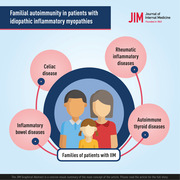

## Introduction

Idiopathic inflammatory myopathies (IIM) are rare rheumatic inflammatory diseases (RIDs) affecting primarily proximal muscles and frequently accompanied by manifestations in other organs. It has been suggested that the pathogenesis of IIM involves both genetic and environmental factors [1]. Numerous genetic variants associated with IIM have been identified, with alleles in the human leukocyte antigen (HLA) region showing the strongest associations [2, 3, 4, 5]. In a recent study, using Swedish family data, we reported that 22% of the phenotypic variance in IIM can be explained by additive genetic variance [6]. This estimate is higher than the single nucleotide polymorphisms based heritability for polymyositis (PM, 8%) and dermatomyositis (DM, 6%) [7], suggesting that some genetic variants associated with IIM are yet to be identified.

When increase in sample size is not feasible, candidate gene studies and meta‐analysis of summary statistics from genome‐wide association studies (GWASs) among multiple diseases with shared susceptibility can be alternative methods to improve discovery without additional recruitment of individuals [8, 9]. Previous studies applying these methods have successfully detected novel genetic variants associated with IIM [3, 4, 5].

Familial associations between two diseases are good indications of shared genetic susceptibility. Evidence of co‐occurrence of autoimmune diseases (ADs) within families of IIM (familial autoimmunity) is either from small‐scale family‐based studies of patients with IIM or other ADs [10, 11, 12, 13, 14] or from large‐scale family‐based studies based primarily on patients with other ADs [15, 16, 17, 18, 19, 20, 21, 22, 23]. Some of these studies observed higher frequencies of rheumatoid arthritis (RA) in families of IIM [10], or reported significant familial associations of RA, systemic lupus erythematosus (SLE), systemic sclerosis (SSc), type 1 diabetes mellitus (T1DM) and autoimmune thyroid diseases (AITD) with IIM [11, 17, 19, 21, 24] while other studies did not support familial associations for RA, Sjögren's syndrome (SS), SSc, multiple sclerosis (MS), inflammatory bowel diseases (IBD), coeliac disease (CeD) and myasthenia gravis (MG) with IIM [11–13, 15, 16, 18, 20, 22–24].

Given these conflicting findings and the lack of replication studies with IIM as the primary focus, we conducted a family‐based study using nationwide Swedish register data to investigate the familial associations between IIM and various ADs. We did this to provide insight into which ADs may share familial susceptibility with IIM, which may also suggest shared genetic susceptibility.

## Patients and methods

### Study setting and data sources

In Sweden, every citizen has equal access to the tax‐funded healthcare system. The National Patient Register (NPR) has systematically collected inpatient and outpatient data on a nationwide level since 1987 and 2001, respectively. The positive predictive value (PPV) of diagnoses of ADs, including RA, IBD, CeD and MG in the NPR varies between 74% and 96% [25, 26]. The Total Population Register (TPR) holds data on nearly 100% of births and deaths in Sweden and is often the source of comparators in epidemiological research [27]. The Multi‐Generation Register (MGR) uses data derived mainly from the TPR and includes parental information of individuals who were ≤15 years of age in 1947 and alive on 1 January 1961. The ascertainment of parents of individuals born in Sweden in 1952 and afterwards is above 90% [28]. The Prescribed Drug Register (PDR) includes nationwide data on the dispensation of prescribed drugs since mid‐2005 [29]. The Swedish Cancer Register has recorded malignancies since 1958, with a 96% of coverage and a validity close to 100% [30].

### Study population

We identified all patients with prevalent IIM in the NPR by requiring ≥1 inpatient visit with IIM as the main diagnosis between 1997 and 2000 or ≥2 inpatient or nonprimary outpatient visits with IIM as the main or contributory diagnoses between 2001 and 2016. The latter algorithm has been previously found to be robust [31]. We only considered the International Classification of Diseases, tenth revision (ICD‐10) codes M33 and G72.4 from internal medicine, rheumatology, dermatology, neurology or paediatrics departments. IIM was further categorised into DM (M33.1), other IIM (M33.2, M33.9 and G72.4) and juvenile IIM (JIIM, M33 or G72.4 with age at diagnosis ≤18 years). We excluded patients with only contributory diagnoses indicating IIM.

We matched each patient with IIM on sex, birth year and residential area, to up to five individuals, alive and living in Sweden, without IIM, randomly from the TPR using incidence density sampling. We hereafter refer to patients with IIM and their matched individuals without IIM as index individuals. To increase the ascertainment of index individuals' biological first‐degree relatives (FDRs), we only included index individuals who were born after 1931 in Sweden. Via linkage to the MGR using the unique identification number of each index individual, we identified their FDRs (parents, full siblings and offspring) and only included those alive in 1987. Lastly, we excluded index individuals without any FDRs identified in the MGR.

### Ascertainment of ADs

We determined diagnoses of RA, SLE, SSc, SS, other systemic connective tissue diseases (SCTD), MS, IBD, T1DM, AITD, CeD and MG in FDRs by requiring ≥1 main diagnosis of that AD in the NPR. To increase the number of cases, we extended the time period for ascertainment to 1987 until 2017 in FDRs. We grouped SS, SLE, SSc and other SCTD into one category called other RIDs, given their overlaps in clinical presentations [1]. We treated RA as an independent entity, given its commonness among ADs [32]. For T1DM, the age at diagnosis should be ≤30 years [33]. As most patients with AITD are treated in primary care and require long‐term thyroid hormone substitution therapy, we also defined an FDR who had ≥1 dispensed prescription of levotyroxinnatrium or liotyroninnatrium in the PDR as having AITD [34]. To examine the robustness of our results, we also applied stricter definitions where we required ≥1 inpatient visit with the main diagnosis of an AD between 1997 and 2001 and ≥2 inpatient or outpatient visits with that AD as the main or contributory diagnoses at specific clinics in the NPR. We did not consider an FDR with only contributory diagnoses of an AD as exposed. We also defined ADs in index individuals and IIM in FDRs using the main definitions. We have described the definitions of each AD in detail in Table S1.

### Statistical methods

We described the basic characteristics and family structure of the index individuals by IIM status. Variables are presented as median with interquartile range (IQR) or frequency with proportion, as appropriate. Differences in prevalence of ADs were tested by chi‐square tests.

We used conditional logistic regression to estimate the odds ratio of familial association by treating IIM in index individuals as the outcome and an AD in FDRs as the exposure. We first computed the odds of having ≥1 and the odds of having ≥2 FDR(s) affected by an AD in patients with IIM compared to individuals without IIM, to explore the potential dose–response relationship between family history of ADs and IIM. Secondly, we treated each FDR pair as an independent unit and analysed familial associations using a structural approach where causal relationships between the disease status of IIM and an AD, individual and familial latent factors in an FDR pair were depicted in directed acyclic graphs (see supplementary methods for details) [35]. In the absence of a causal relationship between IIM and an AD, this approach allowed us to attribute the observed familial association to familial factors shared within FDR pairs, as well as to increase statistical power [35]. In the second modelling method, we used a robust sandwich variance estimator for the standard errors to control for family clustering and additionally adjusted for sex and birth year of FDRs. We also performed subgroup analysis by kinship. If the number of exposed was less than five, we just presented the frequency with proportion.

To test the robustness of the main definitions of ADs, we repeated the analyses using stricter definitions. The second modelling method used in the main analyses assumed no causal relationship between IIM and an AD. Although violation of this assumption is unlikely [36], to further examine the robustness of our findings, we performed two additional analyses under the scenario of an AD causing IIM and the scenario of IIM causing an AD (see supplementary document for details). In the former scenario, we additionally adjusted for the studied AD in index individuals while in the latter one, we additionally adjusted for IIM in FDRs. A remaining association after adjustment would further support the existence of shared familial factors [35].

All analyses were performed with SAS, version 9.4 (SAS Institute, Inc). We gained approval to perform this study from the Swedish Ethical Review Authority (2017/2000‐31).

## Results

**Fig. 1:**
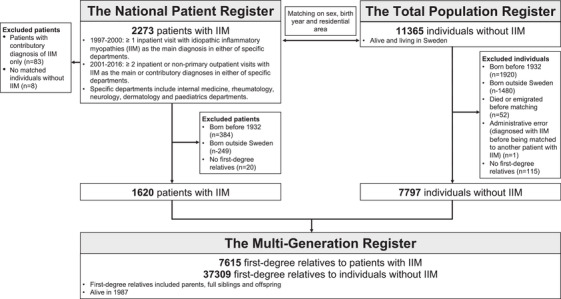
Forming the study population via linkages between different Swedish national registers.

We identified 1620 patients with IIM and 7797 matched individuals without IIM (Fig. 1 and Table 1). Among the index individuals, about 60% were women and the median birth year was 1949. The median age at inclusion was 58 years. Of the 1620 patients with IIM, 8% were diagnosed with JIIM, 31% with DM and 61% with other IIM. Patients with IIM had a significantly higher prevalence of all ADs, except MS, compared to individuals without IIM.

**Table 1:** Characteristics of patients with idiopathic inflammatory myopathies (IIM) and individuals without IIM

	Patients with IIM (*n* = 1620)	Individuals without IIM (*n* = 7797)
Women, *n* (%)	951 (59)	4639 (60)
Birth year, median (Q1–Q3)^a^	1949 (1941–1964)	1949 (1941–1964)
Age at inclusion, median (Q1–Q3)^a^	57 (44–66)	57 (44–65)
IIM subtypes, *n* (%)		
Juvenile IIM^b^	128 (8)	‐
Dermatomyositis^c^	501 (31)	‐
Other IIM^d^	991 (61)	‐
Lifetime autoimmune diseases, *n* (%)		
Rheumatoid arthritis^e^	182 (11)	116 (1)
Rheumatic inflammatory diseases^e^	352 (22)	63 (1)
Multiple sclerosis	5 (0.3)	25 (0.3)
Inflammatory bowel diseases^e^	35 (2)	108 (1)
Type 1 diabetes mellitus^e^	9 (1)	14 (0.2)
Autoimmune thyroid diseases^e^	215 (13)	696 (9)
Coeliac diseases^e^	27 (2)	35 (0.5)
Myasthenia gravis^e^	10 (1)	3 (0.04)

^a^ Q1, the first quartile; Q3, the third quartile.

^b^ Age at diagnosis ≤18 years.

^c^ With diagnostic code M33.1 and age at diagnosis >18 years.

^d^ With diagnostic code M33.2, M33.9 or G72.4 and age at diagnosis >18 years.

^e^ *p‐*values from chi‐square test <0.05.

There were 7615 FDRs to patients with IIM versus 37,309 to individuals without IIM (Table 2). The median number (IQR) of FDRs per index individual was 4 (3–6) for patients with IIM and 5 (3–6) for individuals without IIM.

**Table 2:** Family structures of patients with idiopathic inflammatory myopathies (IIM) and individuals without IIM, and demographic characteristics of their first‐degree relatives^a^

	Patients with IIM		Individuals without IIM	
Any first‐degree relatives, median (Q1–Q3)	7615	4 (3–6)	37,309	5 (3–6)
Parents	2306	2 (1–2)	11,414	2 (1–2)
Women, *n* (%)	1253 (54)		6314 (55)	
Birth year, median (Q1–Q3)	1926 (1916–1943)		1926 (1916–1943)	
Full siblings	2464	1 (0–2)	11,685	1 (0–2)
Women, *n* (%)	1238 (50)		5863 (50)	
Birth year, median (Q1–Q3)	1951 (1943–1962)		1950 (1943–1963)	
Offspring	2845	2 (1–3)	14,210	2 (1–3)
Women, *n* (%)	1335 (47)		6960 (49)	
Birth year, median (Q1–Q3)	1975 (1966–1987)		1975 (1967–1989)	

^a^ Q1, the first quartile; Q3, the third quartile.

### Familial associations of ADs

As shown in Table 3, patients with IIM had statistically significant higher odds of having ≥1 FDR affected by other RIDs (adjusted odds ratio [aOR] = 1.40, 95% confidence interval [CI] 1.11–1.78) and higher odds of having ≥2 FDRs affected by CeD (aOR = 3.57, 95% CI 1.28–9.92), compared to individuals without IIM. Patients with IIM also had slightly higher prevalence of having ≥1 FDR affected by RA, MS, IBD, AITD and MG than individuals without IIM, but none of these associations were statistically significant. A trend of stronger familial association as the number of affected FDRs increased was noted for other RIDs and CeD, although without statistical significance. The dose–response relationships for other ADs were not observed or not studied due to the small number of cases. In the overall analyses, no familial association with T1DM was noted.

**Table 3:** Adjusted odds ratios (aORs) of familial associations between idiopathic inflammatory myopathies (IIM) and different autoimmune diseases by the number of affected first‐degree relatives

	Patients with IIM, *n* (%)	Individuals without IIM, *n* (%)	aOR^a^ (95% CI)
Rheumatoid arthritis			
≥1 First‐degree relative	104 (6.42)	432 (5.54)	1.14 (0.91–1.43)
≥2 First‐degree relatives	6 (0.37)	27 (0.35)	1.10 (0.45–2.70)
Other rheumatic inflammatory diseases^b^			
≥1 First‐degree relative	98 (6.05)	341 (4.37)	1.40 (1.11–1.78)
≥2 First‐degree relatives	6 (0.37)	12 (0.15)	2.40 (0.88–6.53)
Multiple sclerosis			
≥1 First‐degree relative	23 (1.42)	105 (1.35)	1.12 (0.70–1.78)
≥2 First‐degree relatives	1 (0.06)	1 (0.01)	‐
Inflammatory bowel diseases			
≥1 First‐degree relative	98 (6.05)	404 (5.18)	1.23 (0.97–1.55)
≥2 First‐degree relatives	6 (0.37)	29 (0.37)	1.10 (0.44–2.71)
Type 1 diabetes mellitus			
≥1 First‐degree relative	23 (1.42)	109 (1.40)	1.01 (0.64–1.60)
≥2 First‐degree relatives	1 (0.06)	4 (0.05)	‐
Autoimmune thyroid diseases			
≥1 First‐degree relative	424 (26.17)	1877 (24.07)	1.12 (0.99–1.27)
≥2 First‐degree relatives	72 (4.44)	327 (4.19)	1.08 (0.83–1.41)
Coeliac disease			
≥1 First‐degree relative	39 (2.41)	148 (1.90)	1.32 (0.92–1.90)
≥2 First‐degree relatives	7 (0.43)	8 (0.10)	3.57 (1.28–9.92)
Myasthenia gravis			
≥1 First‐degree relative	6 (0.37)	18 (0.23)	1.48 (0.57–3.80)
≥2 First‐degree relatives	0 (0)	0 (0)	‐

Abbreviation: CI, Confidence interval.

^a^ Adjusting for matching factors including sex, birth year and residential area of index individuals. Odds ratios were not presented for cases <5.

^b^ Other rheumatic inflammatory diseases include Sjögren's syndrome, systemic lupus erythematosus, systemic sclerosis and other systemic connective tissue diseases.

The findings of the second modelling method are in line with those resulting from the first modelling method (Table 4). Moreover, significant familial associations were detected for IBD (aOR = 1.20, 95% CI 1.02–1.41) and AITD (aOR = 1.10, 95% CI 1.02–1.19).

**Table 4:** Adjusted odds ratios (aORs) of familial associations between idiopathic inflammatory myopathies (IIM) and different autoimmune diseases in any first‐degree relative pairs and by kinship

	Patients with IIM, *n* (%)	Individuals without IIM, *n* (%)	aOR^a^ (95% CI)
Rheumatoid arthritis			
Any first‐degree relatives	110 (1.44)	462 (1.24)	1.14 (0.97–1.33)
Parents	41 (1.78)	209 (1.83)	0.98 (0.75–1.29)
Full siblings	50 (2.03)	181 (1.55)	1.22 (0.98–1.52)
Offspring	19 (0.67)	72 (0.51)	1.26 (0.84–1.89)
Other rheumatic inflammatory diseases^b^			
Any first‐degree relatives	104 (1.37)	354 (0.95)	1.34 (1.14–1.56)
Parents	46 (1.99)	151 (1.32)	1.47 (1.15–1.89)
Full siblings	35 (1.42)	143 (1.22)	1.04 (0.80–1.35)
Offspring	23 (0.81)	60 (0.42)	1.92 (1.35–2.71)
Multiple sclerosis			
Any first‐degree relatives	24 (0.32)	106 (0.28)	1.19 (0.86–1.65)
Parents	2 (0.09)	14 (0.12)	‐
Full siblings	9 (0.37)	49 (0.42)	0.97 (0.55–1.73)
Offspring	13 (0.46)	43 (0.30)	1.66 (1.10–2.52)
Inflammatory bowel diseases			
Any first‐degree relatives	104 (1.37)	435 (1.17)	1.20 (1.02–1.41)
Parents	29 (1.26)	102 (0.89)	1.41 (1.02–1.94)
Full siblings	29 (1.18)	155 (1.33)	0.92 (0.66–1.28)
Offspring	46 (1.62)	178 (1.25)	1.30 (1.01–1.68)
Type 1 diabetes mellitus			
Any first‐degree relatives	24 (0.32)	113 (0.30)	1.10 (0.77–1.55)
Parents	0 (0)	5 (0.04)	‐
Full siblings	7 (0.28)	26 (0.22)	1.66 (0.77–3.57)
Offspring	17 (0.60)	82 (0.58)	1.13 (0.74–1.72)
Autoimmune thyroid diseases			
Any first‐degree relatives	509 (6.68)	2281 (6.11)	1.10 (1.02–1.19)
Parents	171 (7.42)	829 (7.26)	0.99 (0.86–1.14)
Full siblings	200 (8.12)	882 (7.55)	1.06 (0.93–1.20)
Offspring	138 (4.85)	570 (4.01)	1.20 (1.03–1.39)
Coeliac disease			
Any first‐degree relatives	47 (0.62)	156 (0.42)	1.37 (1.08–1.74)
Parents	6 (0.26)	15 (0.13)	2.29 (1.22–4.29)
Full siblings	19 (0.77)	58 (0.50)	1.45 (1.01–2.09)
Offspring	22 (0.77)	83 (0.58)	1.28 (0.90–1.83)
Myasthenia gravis			
Any first‐degree relatives	6 (0.08)	18 (0.05)	1.45 (0.77–2.74)
Parents	3 (0.13)	12 (0.11)	‐
Full siblings	3 (0.12)	4 (0.03)	‐
Offspring	0 (0)	2 (0.01)	‐

Abbreviation: CI, Confidence interval.

^a^ Additionally adjusting for sex and birth year of first‐degree relatives. Odds ratios were not presented for cases <5.

^b^ Other rheumatic inflammatory diseases include Sjögren's syndrome, systemic lupus erythematosus, systemic sclerosis and other systemic connective tissue diseases.

In the subgroup analyses, we found that the familial associations for each AD were not consistent between parents, full siblings and offspring (Table 4). Familial associations were only found in parents and in offspring but not in full siblings for other RIDs and IBD, while familial associations of MS and AITD were detected only in offspring. Moreover, familial associations of CeD were more apparent in parents and in full siblings. There were also signals of familial associations of RA and T1DM in full siblings and in offspring, but they were not statistically significant. We did not perform subgroup analyses for MG due to the small number of cases.

### Sensitivity analyses

Using stricter definitions to define ADs resulted in similar point estimates as those of the main analyses (Table S2). Additional adjustment of an AD in index individuals or IIM in FDRs generally did not significantly affect the findings (Table S3). Specifically, we found similar point estimates of aORs but lost significance for other RIDs, IBD and AITD after adjusting for them respectively in index individuals.

## Discussion

In this population‐based family study, findings from the two statistical modelling methods together suggest that family history of other RIDs, IBD, AITD and CeD in FDRs was significantly associated with IIM. The familial associations between IIM and other RIDs and CeD were further supported by the fact that there was a dose–response relationship between IIM and these ADs; the familial association increased with the number of affected FDRs. For MS, unlike other RIDs, IBD, AITD and CeD, which showed significant familial associations with IIM not only in the overall analysis but also in at least one type of kinship, we only observed familial association among offspring. For RA, T1DM and MG, we detected no significant findings though a signal of a familial association was noted in the stratified analysis by kinship for RA and TIDM and in the overall analysis for MG. The main and the sensitivity analyses provided consistent findings, suggesting the robustness of our findings. Together, these findings might imply shared familial susceptibility between IIM and various ADs, in particular other RIDs and CeD, which was potentially attributable to both genetic and environmental factors.

Previous small‐scale family studies observed higher frequencies of RA, SLE and AITD and T1DM in families of patients with IIM, but their findings are inconsistent [10, 11, 14]. Moreover, it is difficult to compare our findings to those of these studies due to differences in study design, study populations and data sources.

Family‐based studies using population data are more comparable to ours, though the majority of relevant studies had a focus on patients with other ADs. In the study of Thomsen et al., also using Swedish nationwide register data, familial associations were observed for RA (standardised incidence ratio [SIR] = 1.35, 95% CI 1.06–1.68) and SLE (SIR = 2.28, 95% CI 1.06–1.68) in 1384 patients with PM/DM [24]. Another Swedish study of Graves' disease reported familial association with PM/DM (SIR = 2.48, 95% CI 1.12–4.73) [17]. Two population‐based family studies using insurance data in Taiwan observed that individuals with FDRs affected by IIM had about threefold higher risk of developing SLE [19] and an eightfold increased risk of having SSc [21]. These stronger familial associations compared to ours for other RIDs and AITD might be due to the inclusion of only hospitalised patients or patients insured for catastrophic illness in those studies, which are usually associated with more severe disease status [17, 19, 21].

There are some population‐based or large‐scale family studies that did not find any significant familial associations between IIM and RA, SSc, SS, MS and IBD [15, 16, 18, 20, 22–24]. Differences between ethnic populations could be one reason. A Taiwanese study of patients with RA found no familial association with IIM [23] while a Swedish study did [24]. Another Swedish study also pointed towards an association although without statistical significance [22]. Insufficient power might also explain some of the discrepancies compared to our findings. For example, a study including 1071 patients with SSc found no familial association with IIM whereas Kuo et al. did in a study including 1891 patients with SSc [16, 21]. Given the rarity of IIM, a sufficiently large sample of patients with other ADs may be required to detect familial association with IIM, otherwise the potential association may be missed. Even in a study including 25,846 patients with ulcerative colitis, there were only four FDRs affected by IIM [18]. Our study consisted of a large sample of patients with IIM to achieve good statistical precision. As familial association also involves environmental factors shared within families, exposure to different environmental factors between countries could be another reason why our results differ from previous studies. For example, smoking is associated with many ADs, including IIM [1, 37]. Smoking habit is likely to be shared within families but the sharing pattern may differ between countries as there is a huge variation in smoking prevalence around the world [38]. However, we have no data on environmental factors, making it difficult to explore this further. In addition, replication studies are needed to confirm our findings, especially for TIDM, CeD and MG, with no prior population‐based studies exploring their familial associations with IIM, and for MS with significant association observed only in offspring.

Another interesting finding in our study is that familial associations between IIM and ADs varied among parents, full siblings and offspring. Variation in ascertainment of ADs between parents, full siblings and offspring due to births in different calendar periods could be one explanation (Table 2). For example, inpatient data might be weighted more in the ascertainment of ADs in parents than in full siblings and offspring. Therefore, we might ascertain more severe cases of ADs in parents, which might affect the validity of estimation. Furthermore, offspring had the shortest follow‐up time in adulthood and thus the observed familial associations of ADs might likely represent cases of ADs with early onset. The familial associations of other RIDs, MS, IBD and AITD in offspring might suggest genetic contribution. Compared to parents and offspring, full siblings had more completed coverage on both inpatient and outpatient care and the longest follow‐up time. The familial associations of ADs in full siblings were the estimates with more completed coverage of ADs. In our study, only CeD showed significant familial association among full siblings. All FDRs share, on average, 50% of their genetics. Full siblings also share environmental factors growing up, something that sets them apart from parent–offspring pairs. This means that shared environmental factors, besides shared genetic factors, might be more important to explain the familial association for CeD than other ADs. However, interpretation of these findings should be made with caution as the observed variations could also be caused by unobserved heterogeneity arising from randomness, statistical modelling and/or other unknown factors [39].

For complex diseases, an observed familial association tells us little about the disease's inheritance pattern and the extent of genetic contribution to disease development [40]. Nevertheless, the value of evidence of familial autoimmunity in guiding genetic research and in understanding the disease pathogenesis of complex diseases should not be disregarded. For example, two genetic studies of IIM were guided by prior knowledge of the individual and familial associations observed between IIM and different ADs, particularly RIDs [4, 5]. A candidate gene study within a GWAS testing the associations of 141 AD‐associated genetic markers outside the HLA region with DM discovered three new genetic variants [5]. Another study meta‐analysing GWAS data of IIM, SLE, SSc and RA found four novel genetic loci associated with IIM [4]. Compared to other RIDs, the association between IIM and CeD is less recognised but increased prevalence of CeD in patients with IIM [11, 41], shared pathogenic components [1, 42] and HLA alleles with IIM (HLA‐DQB1*02 and HLA‐DQA1*05:01) [2, 42] have been reported. Our findings may further add to the evidence suggesting shared pathogenesis between IIM and CeD and genetics could be the contributing factors. Unfortunately, our data sources did not allow for the quantification of genetic contribution. As GWAS data on many ADs are available for secondary analysis, GWAS meta‐analysis of IIM and CeD may help to answer this research question and may also boost GWAS discovery [43, 44].

Our findings also have clinical implications. Family history of ADs is one of the indicators towards diagnosis of IIM [1]. This knowledge is however not specified to which and to what extent ADs have familial associations with IIM. Our findings shrink this knowledge gap and suggest that suspected cases of IIM with family history of other RIDs, MS, IBD, AITD and CeD among FDRs might need more attention, especially for those with ≥2 FDRs affected by CeD.

Our study has several limitations. Although ascertainment of RA, SLE, SS, MS, IBD, T1DM, CeD and MG using ≥1 main diagnosis in the NPR was with high validity, with PPV ranging from 74% to 97% [25, 26, 33, 45, 46], the PPV of SSc (68%) was lower [47]. Such misclassification of SSc, in particular, could potentially lead to familial associations being diluted toward null. However, we minimised this risk by grouping SSc with other RIDs. Moreover, using only data from the NPR cannot ascertain cases of CeD treated only in primary care, which is the general case in Sweden [48]. This underdetection might be more problematic in families of the general population than in families of patients with IIM, where greater awareness to seek specialist healthcare was likely. Therefore, we might be overestimating the familial association between IIM and CeD.

Further, previous findings suggest that the types of ADs showing familial associations with IIM seemed to be different between adult IIM and JIIM [10, 11, 14]. For example, higher prevalence of family history of T1DM was only found in studies of juvenile dermatomyositis [11, 14]. We could not perform analyses by age at diagnosis of IIM due to insufficient power. This might also explain why we did not observe familial association between IIM and T1DM as the majority of patients included in our study were adult IIM. Another problem associated with T1DM was that we only had data from the NPR since 1987. Those FDRs, especially parents and full siblings who were born in earlier years, might thus have been defined as unexposed even they actually had T1DM if the age at the first record of T1DM in the NPR was >30 years. This bias might dilute the familial association of T1DM. The age at diagnosis of ADs could be another factor affecting our estimations. For example, the genetic susceptibility of early‐onset MG is mainly explained by the HLA region while alleles outside the HLA region in addition to HLA alleles confer risk of late‐onset MG [49, 50]. We also could not perform analyses by age at onset of ADs because of the small number of cases. Lastly, the generalisability of our findings might be limited to Caucasian populations as polymorphisms associated with ADs differ between ethnicities [51].

Despite these limitations, our study is the first population‐based family study comprehensively examining the familial associations between IIM and various ADs. Using prospectively collected register data with high validity, we included a large representative sample of patients with IIM and minimised bias of self‐reported data observed in previous studies. Furthermore, with the matching design, biases due to factors like family structure, sex and birth year of the study population were minimised. We also performed a number of sensitivity analyses and showed that our findings were robust to the change of definitions of ADs and to the bias rising from additional adjustment of IIM or an AD.

In summary, IIM like other ADs, shows familial autoimmunity. The associated ADs besides other RIDs were MS, IBD, AITD and CeD. This information is useful to clinical counselling, and to guide future genetic studies of IIM.

## Author contributions

Weng Ian Che: Conceptualization; Data curation; Formal analysis; Investigation; Methodology; Project administration; Software; Validation; Visualization; Writing – original draft; Writing – review and editing. Helga Westerlind: Conceptualization; Data curation; Formal analysis; Investigation; Methodology; Supervision; Validation; Writing – review and editing. Ingrid E. Lundberg: Conceptualization; Funding acquisition; Investigation; Methodology; Supervision; Validation; Writing – review and editing. Karin Hellgren: Conceptualization; Investigation; Methodology; Supervision; Validation; Writing – review and editing. Ralf Kuja‐Halkola: Conceptualization; Formal analysis; Investigation; Methodology; Supervision; Validation; Writing – review and editing. Marie E. Holmqvist: Conceptualization; Data curation; Funding acquisition; Investigation; Methodology; Resources; Supervision; Validation; Writing – original draft; Writing – review and editing.

## Conflict of interest

I.E.L. and M.H. have received grants from the Swedish Research Council. M.H. is a member of the medical advisory board of the Myositis Association. The other authors declare no conflict of interest.

## Supporting information




**Supplementary Figure 1**. The proposed underlying mechanisms between idiopathic inflammatory myopathies (IIM) and an autoimmune disease (AD) in a Direct Acyclic Graph.
**Supplementary Table 1**. Definitions of idiopathic inflammatory myopathies (IIM) and other autoimmune diseases (ADs) in first‐degree relatives.
**Supplementary Table 2**. Adjusted odds ratios (aORs) of familial associations between idiopathic inflammatory myopathies (IIM) and different autoimmune diseases using stricter definitions.
**Supplementary Table 3**. Adjusted odds ratios (aORs) of familial associations between idiopathic inflammatory myopathies (IIM) and different autoimmune diseases, additionally adjusted for each autoimmune disease in index persons and IIM in first‐degree relatives.
