# Supplementary material for: Familial autoimmunity in patients with idiopathic inflammatory myopathies
Source: J Intern Med. 2022 Oct 10;293(2):200–11. doi: 10.1111/joim.13573 (PMC10092836; doi:10.1111/joim.13573)
Supplement: Supplementary file 1 — Supplementary Figure 1. The proposed underlying mechanisms between idiopathic inflammatory myopathies (IIM) and an autoimmune disease (AD) in a Direct Acyclic Graph. Supplementary Table 1. Definitions of idiopathic inflammatory myopathies (IIM) and other autoimmune diseases (ADs) in first‐degree relatives. Supplementary Table 2. Adjusted odds ratios (aORs) of familial associations between idiopathic inflammatory myopathies (IIM) and different autoimmune diseases using stricter definitions. Supplementary Table 3. Adjusted odds ratios (aORs) of familial associations between idiopathic inflammatory myopathies (IIM) and different autoimmune diseases, additionally adjusted for each autoimmune disease in index persons and IIM in first‐degree relatives. [file JOIM-293-200-s001.docx]

**Familial autoimmunity in patients with idiopathic inflammatory myopathies: Supplementary methods and data**

Weng Ian Che^1^, Helga Westerlind^1^, Ingrid E. Lundberg^2,3^, Karin Hellgren^1,2^, Ralf Kuja-Halkola^4^, Marie Holmqvist^1,2^

^1^Clinical epidemiology division, Department of Medicine, Solna, Karolinska Institutet, Stockholm, Sweden.

^2^Division of Rheumatology, Department of Medicine, Solna, Karolinska Institutet, Stockholm, Sweden.

^3^ME Gastro, Derm and Rheuma, Theme Inflammation and Aging, Karolinska University Hospital, Stockholm, Sweden. ^4^Department of Medical Epidemiology and Biostatistics, Karolinska Institutet, Stockholm, Sweden.

**Familial associations between idiopathic inflammatory myopathies and other autoimmune diseases under three potential underlying mechanisms using a structural approach with Directed Acyclic Graphs**

According to the method proposed by Hudson *et al.*, we used Directed Acyclic Graphs (DAGs) to analyse familial association between idiopathic inflammatory myopathies (IIM) and an autoimmune disease (AD) under different potential pathological mechanisms between the two diseases [1]. The principles of DAG in epidemiological research have been described in detail elsewhere [2]. In this study, for each index person-relative pair, as depicted in supplementary figure 1a, a DAG begins with four disease variables indicating IIM status and an AD status in the index person (IIM_1_ and AD_1_) and in the first-degree relative (FDR) (IIM_2_ and AD_2_), respectively. The latent variable C represents shared familial factors between IIM and an AD thus there are four directed edges pointing from C to each disease variable depicting the causal relationships between C and the four disease variables. Besides the latent variable C, there are other latent variables causing IIM and an AD in the same individual or causing either IIM or AD across individuals in the relative pair. U_1_ and U_2_ represent unique individual factors that can lead to both IIM and an AD in the index person and in the FDR, respectively. For example, U_1/2_ can be sex or birth year of the index person or the FDR. U_IIM_ and U_AD_ indicate factors unique to each disease that are not shared within families. As shown in all three DAGs in supplementary figure 1, we assume that all latent variables are independent to each other and have additive effects.

**Supplementary figure 1. The proposed underlying mechanisms between idiopathic inflammatory myopathies (IIM) and an autoimmune disease (AD) in a Direct Acyclic Graph.** IIMj and ADj represent IIM and an AD respectively, for individual j in a given pair; j = 1, 2. U_IIM_ represents common causes for IIM_1_ and IIM_2_, and U_AD_ represents common causes for AD_1_ and AD_2_. Uj represents unique individual common causes for IIMj and ADj that may vary within the pair, and C represents shared family factors for IIMj and ADj that are constant within the pair. a. No causal relationship; b. Causal relationship from an AD to IIM; c. Causal relationship from IIM to an AD.

IIM_1_

IIM_2_

AD_1_

AD_2_

C

U_IIM_

U_AD_

U_1_

U_2_

IIM_1_

IIM_2_

AD_1_

AD_2_

C

U_IIM_

U_AD_

U_1_

U_2_

IIM_1_

IIM_2_

AD_1_

AD_2_

C

U_IIM_

U_AD_

U_1_

U_2_

**a**

**b**

**c**

The DAG in supplementary figure 1a assumes that there are no causal relationships between IIM and an AD. Under this assumption, IIM_1_🡨C🡪AD_2_ is the only open path (or estimated statistical association) in the DAG and we can interpret that the observed familial association between IIM and an AD is caused by C, the shared familial factors [1].

Though there is little evidence supporting causality between IIM and the studied ADs, we analyse familial associations between IIM and other ADs under the presence of causal relationships between diseases as sensitivity analyses to test the robustness of our findings.

We firstly assume that there is a causal relationship from an AD to IIM, as shown in supplementary figure 1b. After introducing this relationship, two paths that were previously closed are now open (IIM_1_🡨AD_1_🡨C🡪AD_2_ and IIM_1_🡨AD_1_🡨U_AD_🡪AD_2_) beside the path IIM_1_🡨C🡪AD_2_. We expect that all these paths represent positive associations between IIM_1_ and AD_2_ however the path IIM_1_🡨AD_1_🡨U_AD_ 🡪AD_2_ does not include C. Therefore, when this causal effect from an AD to IIM presents and it is ignored, we may overestimate the familial association between IIM and the AD, and we can consider this estimate as the upper bound of familial association. We may control for this bias by adjusting for the AD in the index person. After that, the two paths (IIM_1_🡨AD_1_🡪C 🡪AD_2_ and IIM_1_🡨AD_1_🡨U_AD_🡪AD_2_) are closed but we open three additional paths that are previously blocked (IIM_1_🡨U_1_🡪AD_1_🡨U_AD_🡪AD_2_, IIM_1_🡨U_1_🡪AD_1_🡨C🡪AD_2_ and IIM_1_🡨C🡪AD_1_🡨U_AD_🡪AD_2_). Moreover, AD_1_ is a collider for U_1_ and C and for U_AD_ and C. Adjusting for AD_1_ can lead to collider-stratification bias and this bias is likely to be negative. It is because U_1_ and C, and U_AD_ and C are likely to affect AD_1_ in the same direction [3]. Therefore, adjustment of AD_1_ may introduce downward bias to the estimation of familial association, and we can consider the estimate after adjustment as the lower bound of association. If the estimate remains significant after adjustment, it is strong evidence of existence of shared familial factors between IIM and the AD [1].

In supplementary figure 1c, we assume that there is causal relationship from IIM to an AD. As the concepts are similar to those described above, we simplify the description. Under this assumption, there are three open paths before adjustment (IIM_1_🡨C🡪AD_2_, IIM_1_🡨C🡪 IIM_2_🡪AD_2_ and IIM_1_🡨U_IIM_🡪IIM_2_🡪AD_2_). To control for the bias under this causal relationship assumption, we can adjust for IIM in the FDR to close the two latter paths, but this can lead to opening of three additional paths (IIM_1_🡨C🡪IIM_2_🡨U_2_🡪AD_2_, IIM_1_🡨U_IIM_🡪IIM_2_🡨C🡪AD_2_ and IIM_1_🡨U_IIM_🡪IIM_2_🡨U_2_🡪AD_2_). This can result in downward bias of familial association and again, significant estimate after adjustment further supports the existence of shared familial factors between IIM and the AD.

Taken together, the purpose of doing these analyses is to provide further evidence to support the existence of shared familial factors between IIM and other ADs if the statistical significance remains after adjustment.

| **Supplementary table 1. Definitions of idiopathic inflammatory myopathies (IIM) and other autoimmune diseases (ADs) in first-degree relatives** | | | | | |
| --- | --- | --- | --- | --- | --- |
| **ADs** | **Time period** | **ICD codes** | **Types of diagnosis** | **Main definition** | **Stricter definition** |
| IIM | 1987-2017 | 710D, 710E, M33.0, M33.1, M33.2, M33.9, G72.4 | Main/contributory diagnosis | ≥ 1 main diagnosis in the NPR | ≥ 1 main diagnosis in inpatient register between 1987 and 2000 or ≥ 2 diagnoses (≥ 1 main diagnosis) in the NPR between 2001 and 2017 in internal medicine, rheumatology, neurology, dermatology or paediatric clinic |
| Rheumatoid arthritis | 1987-2017 | 714A-C, 714W, M05, M06.0, M06.2, M06.3, M06.8, M06.9 | Main/contributory diagnosis | ≥ 1 main diagnosis in the NPR | ≥ 1 main diagnosis in inpatient register between 1987 and 2000 or ≥ 2 diagnoses (≥ 1 main diagnosis) in the NPR between 2001 and 2017 in internal medicine, rheumatology or paediatric clinic |
| Other rheumatic inflammatory diseases | 1987-2017 | 710C, M35, 710A, M32, 710B, M34.0, M34.1, M34.8 and M34.9,  136B, 725, 710W, 710X, | Main diagnosis | ≥ 1 main diagnosis in the NPR | - |
| Multiple sclerosis | 1987-2017 | 340, G35 | Main/contributory diagnosis | ≥ 1 main diagnosis in the NPR | ≥ 1 main diagnosis in inpatient register between 1987 and 2000 or ≥ 2 diagnoses (≥ 1 main diagnosis) in the NPR between 2001 and 2017 in internal medicine or neurology or paediatrics clinic |
| Inflammatory bowel diseases | 1987-2017 | 555, 556, K50-51 | Main/contributory diagnosis | ≥ 1 main diagnosis in the NPR | ≥ 1 main diagnosis in inpatient register between 1987 and 2000 or ≥ 2 diagnoses (≥ 1 main diagnosis) in the NPR between 2001 and 2017 in internal medicine or gastroenterology or rheumatology or surgical care or gastrointestinal care or paediatrics clinic |
| Type 1 diabetes mellitus | 1987-2017 | 250, E10 | Main/contributory diagnosis | ≥ 1 main diagnosis in the NPR. The diagnosis had to be made ≤30 years of age as the ICD code 250 cannot distinguish T1DM from T2DM and E10 might be used for T2DM as it can developed into insulin dependency. | ≥ 1 main diagnosis in inpatient register between 1987 and 2000 or ≥ 2 diagnoses (≥ 1 main diagnosis) in the NPR between 2001 and 2017 in endocrinology or internal medicine or paediatrics clinic. The diagnosis had to be made ≤30 years of age as this ICD code 250 cannot distinguish T1DM from T2DM and E10 might be used for T2DM as it can developed into insulin dependency. |
| Autoimmune thyroid diseases | 1987-2017 | E038, E039, E063, 244x, 245C; E050, E051, E052, E053, E058, E059, 242, 242A, 242B, 242D, 242E; *O905* | Main diagnosis | ≥ 1 main diagnosis in the NPR or ≥1 filling of a prescription of thyroid hormone substitution therapy (H03AA01 and H03AA02) between 2005-2017, with no history of thyroid cancer or a prescription of iodine-containing drugs (C01BD01, N05AN01, L03AB01/L03AB04/L03AB05) | ~~-~~ |
| Celiac disease | 1987-2017 | 579A, K90.0 | Main/contributory diagnosis | ≥ 1 main diagnosis in the NPR | ≥ 1 main diagnosis in inpatient register between 1987 and 2000 or ≥ 2 diagnoses (≥ 1 main diagnosis) in the NPR between 2001 and 2017 in internal medicine, gastroenterology, gastrointestinal care, paediatrics clinic |
| Myasthenia gravis | 1987-2017 | 358A, G70.0 | Main/contributory diagnosis | ≥ 1 main diagnosis in the NPR | ≥ 1 main diagnosis in inpatient register between 1987 and 2000 or ≥ 2 diagnoses (≥ 1 main diagnosis) in the NPR between 2001 and 2017 in internal medicine or neurology or paediatrics clinic |

| **Supplementary table 2. Adjusted odds ratios (aORs) of familial associations between idiopathic inflammatory myopathies (IIM) and different autoimmune diseases using stricter definitions** | | | |  |
| --- | --- | --- | --- | --- |
|  | **Patients with IIM, n (%)** | **Individuals without IIM, n (%)** | **aOR^a^ (95% CI)** | |
| **Rheumatoid arthritis** |  |  |  | |
| ≥1 first-degree relative | 81 (5.00) | 341 (4.37) | 1.13 (0.88-1.46) | |
| ≥2 first-degree relatives | 5 (0.31) | 21 (0.27) | 1.16 (0.43-3.11) | |
| Any first-degree relatives | 86 (1.13) | 365 (0.98) | 1.12 (0.94-1.33) | |
| Parents | 32 (1.39) | 155 (1.36) | 1.00 (0.74-1.36) | |
| Full siblings | 38 (1.54) | 153 (1.31) | 1.14 (0.89-1.47) | |
| Offspring | 16 (0.56) | 57 (0.40) | 1.39 (0.89-2.15) | |
| **Multiple sclerosis** |  |  |  | |
| ≥1 first-degree relative | 19 (1.17) | 92 (1.18) | 1.08 (0.65-1.79) | |
| ≥2 first-degree relatives | 0 (0) | 1 (0.01) | - | |
| Any first-degree relatives | 19 (0.25) | 93 (0.25) | 1.10 (0.77-1.57) | |
| Parents | 2 (0.09) | 12 (0.11) | - | |
| Full siblings | 7 (0.28) | 43 (0.37) | 0.84 (0.45-1.57) | |
| Offspring | 10 (0.35) | 38 (0.27) | 1.47 (0.93-2.33) | |
| **Inflammatory bowel diseases** |  |  |  | |
| ≥1 first-degree relative | 77 (4.75) | 326 (4.18) | 1.19 (0.92-1.55) | |
| ≥2 first-degree relatives | 5 (0.31) | 23 (0.29) | 1.24 (0.45-3.36) | |
| Any first-degree relatives | 82 (1.08) | 350 (0.94) | 1.20 (0.99-1.44) | |
| Parents | 22 (0.95) | 83 (0.73) | 1.34 (0.93-1.94) | |
| Full siblings | 23 (0.93) | 122 (1.04) | 0.95 (0.65-1.38) | |
| Offspring | 37 (1.30) | 145 (1.02) | 1.32 (0.99-1.75) | |
| **Type 1 diabetes mellitus** |  |  |  | |
| ≥1 first-degree relative | 22 (1.36) | 105 (1.35) | 1.02 (0.64-1.62) | |
| ≥2 first-degree relatives | 1 (0.06) | 4 (0.05) | **-** | |
| Any first-degree relatives | 23 (0.30) | 109 (0.29) | 1.10 (0.77-1.58) | |
| Parents | 0 (0) | 5 (0.04) | - | |
| Full siblings | 6 (0.24) | 25 (0.21) | 1.52 (0.64-3.63) | |
| Offspring | 17 (0.60) | 79 (0.56) | 1.21 (0.79-1.83) | |
| **Celiac disease** |  |  |  | |
| ≥1 first-degree relative | 26 (1.60) | 89 (1.14) | 1.45 (0.92-2.29) | |
| ≥2 first-degree relatives | 4 (0.25) | 7 (0.09) | - | |
| Any first-degree relatives | 30 (0.39) | 94 (0.25) | 1.48 (1.12-1.97) | |
| Parents | 4 (0.17) | 11 (0.10) | - | |
| Full siblings | 12 (0.49) | 28 (0.24) | 1.80 (1.21-2.69) | |
| Offspring | 14 (0.49) | 55 (0.39) | 1.27 (0.79-2.04) | |
| **Myasthenia gravis** |  |  |  | |
| ≥1 first-degree relative | 5 (0.31) | 16 (0.21) | 1.37 (0.49-3.83) | |
| ≥2 first-degree relatives | 0 (0) | 0 (0) | - | |
| Any first-degree relatives | 4 (0.05) | 14 (0.04) | - | |
| Parents | 2 (0.09) | 10 (0.09) | - | |
| Full siblings | 2 (0.08) | 3 (0.03) | - | |
| Offspring | 0 (0) | 1 (0.01) | - | |
| a In the analyses by number of affected first-degree relatives, matching factors including sex, birth year and residential area of index individuals were controlled. In the analyses among any first-degree relatives, parents, full siblings and offspring, sex and birth year of first-degree relatives were additionally adjusted. Odds ratios were not presented for cases <5. CI: Confidence Interval. | | | |  |

| **Supplementary table 3. Adjusted odds ratios (aORs) of familial associations between idiopathic inflammatory myopathies (IIM) and different autoimmune diseases, additionally adjusted for each autoimmune disease in index persons and IIM in first-degree relatives** | | | | |
| --- | --- | --- | --- | --- |
|  | **Patients with IIM, n (%)** | **Individuals without IIM, n (%)** | **aOR^a^ (95% CI)** | **aOR^b^ (95% CI)** |
| **Rheumatoid arthritis** |  |  |  |  |
| Any first-degree relatives | 110 (1.44) | 462 (1.24) | 1.02 (0.88-1.20) | 1.13 (0.97-1.32) |
| Parents | 41 (1.78) | 209 (1.83) | 0.89 (0.67-1.17) | 0.98 (0.75-1.28) |
| Full siblings | 50 (2.03) | 181 (1.55) | 1.22 (0.90-1.40) | 1.22 (0.98-1.52) |
| Offspring | 19 (0.67) | 72 (0.51) | 1.09 (0.75-1.58) | 1.26 (0.84-1.89) |
| **Other rheumatic inflammatory diseases^c^** |  |  |  |  |
| Any first-degree relatives | 104 (1.37) | 354 (0.95) | 1.11 (0.96-1.28) | 1.30 (1.12-1.52) |
| Parents | 46 (1.99) | 151 (1.32) | 1.23 (0.95-1.59) | 1.45 (1.13-1.87) |
| Full siblings | 35 (1.42) | 143 (1.22) | 0.93 (0.72-1.19) | 1.02 (0.78-1.33) |
| Offspring | 23 (0.81) | 60 (0.42) | 1.32 (0.92-1.89) | 1.85 (1.30-2.63) |
| **Multiple sclerosis** |  |  |  |  |
| Any first-degree relatives | 24 (0.32) | 106 (0.28) | 1.19 (0.86-1.65) | 1.20 (0.86-1.66) |
| Parents | 2 (0.09) | 14 (0.12) | 0.66 (0.18-2.38) | 0.66 (0.18-2.42) |
| Full siblings | 9 (0.37) | 49 (0.42) | 0.98 (0.55-1.73) | 0.97 (0.55-1.73) |
| Offspring | 13 (0.46) | 43 (0.30) | 1.66 (1.10-2.52) | 1.66 (1.10-2.52) |
| **Inflammatory bowel diseases** |  |  |  |  |
| Any first-degree relatives | 104 (1.37) | 435 (1.17) | 1.17 (1.00-1.38) | 1.20 (1.02-1.41) |
| Parents | 29 (1.26) | 102 (0.89) | 1.42 (1.03-1.95) | 1.41 (1.02-1.94) |
| Full siblings | 29 (1.18) | 155 (1.33) | 0.89 (0.64-1.24) | 0.92 (0.66-1.28) |
| Offspring | 46 (1.62) | 178 (1.25) | 1.27 (0.99-1.64) | 1.30 (1.01-1.68) |
| **Type 1 diabetes mellitus** |  |  |  |  |
| Any first-degree relatives | 24 (0.32) | 113 (0.30) | 1.07 (0.75-1.52) | 1.10 (0.77-1.55) |
| Parents | 0 (0) | 5 (0.04) | - | - |
| Full siblings | 7 (0.28) | 26 (0.22) | 1.62 (0.74-3.56) | 1.65 (0.77-3.56) |
| Offspring | 17 (0.60) | 82 (0.58) | 1.11 (0.73-1.71) | 1.13 (0.74-1.72) |
| **Autoimmune thyroid diseases** |  |  |  |  |
| Any first-degree relatives | 509 (6.68) | 2281 (6.11) | 1.07 (0.99-1.15) | 1.10 (1.01-1.18) |
| Parents | 171 (7.42) | 829 (7.26) | 0.96 (0.83-1.11) | 0.99 (0.86-1.14) |
| Full siblings | 200 (8.12) | 882 (7.55) | 1.02 (0.90-1.16) | 1.05 (0.92-1.20) |
| Offspring | 138 (4.85) | 570 (4.01) | 1.15 (0.99-1.34) | 1.19 (1.02-1.38) |
| **Celiac disease** |  |  |  |  |
| Any first-degree relatives | 47 (0.62) | 156 (0.42) | 1.29 (1.01-1.64) | 1.37 (1.08-1.74) |
| Parents | 6 (0.26) | 15 (0.13) | 2.10 (1.13-3.90) | 2.29 (1.22-4.29) |
| Full siblings | 19 (0.77) | 58 (0.50) | 1.38 (0.95-2.00) | 1.45 (1.01-2.09) |
| Offspring | 22 (0.77) | 83 (0.58) | 1.20 (0.84-1.73) | 1.28 (0.90-1.83) |
| **Myasthenia gravis** |  |  |  |  |
| Any first-degree relatives | 6 (0.08) | 18 (0.05) | 1.24 (0.67-2.30) | 1.44 (0.76-2.73) |
| Parents | 3 (0.13) | 12 (0.11) | - | - |
| Full siblings | 3 (0.12) | 4 (0.03) | - | - |
| Offspring | 0 (0) | 2 (0.01) | - | - |
| a Controlled for sex and birth year of index individuals and first-degree relatives, and the studied autoimmune disease in index persons. Odds ratios were not presented for cases <5. CI: Confidence Interval.  b Controlled for sex and birth year of index individuals and first-degree relatives, and IIM in first-degree relatives. Odds ratios were not presented for cases <5. CI: Confidence Interval.  c Other rheumatic inflammatory diseases include Sjögren’s syndrome, systemic lupus erythematosus, systemic sclerosis and other systemic connective tissue diseases. | | | | |

**References**

1. Hudson JI, Javaras KN, Laird NM, VanderWeele TJ, Pope HG, Jr., Hernan MA. A structural approach to the familial coaggregation of disorders. Epidemiology. 2008;19(3):431-9. doi: 10.1097/EDE.0b013e31816a9de7. PubMed PMID: 18379420.

2. Greenland S, Pearl J, Robins JM. Causal diagrams for epidemiologic research. Epidemiology. 1999;10(1):37-48. PubMed PMID: 9888278.

3. Cole SR, Platt RW, Schisterman EF, Chu H, Westreich D, Richardson D, et al. Illustrating bias due to conditioning on a collider. Int J Epidemiol. 2010;39(2):417-20. doi: 10.1093/ije/dyp334. PubMed PMID: 19926667; PubMed Central PMCID: PMCPMC2846442.
